# Plasma membrane profiling during enterohemorrhagic *E. coli* infection reveals that the metalloprotease StcE cleaves CD55 from host epithelial surfaces

**DOI:** 10.1074/jbc.RA118.005114

**Published:** 2018-09-06

**Authors:** R. Christopher D. Furniss, Wen Wen Low, Despoina A. I. Mavridou, Laura F. Dagley, Andrew I. Webb, Edward W. Tate, Abigail Clements

**Affiliations:** From the ‡ MRC Centre for Molecular Bacteriology and Infection, Department of Life Sciences, Imperial College London, London, SW7 2AZ United Kingdom,; § Walter and Eliza Hall Institute of Medical Research, Melbourne 3052, Australia,; ¶ Department of Medical Biology, University of Melbourne, Melbourne 3050, Australia, and; ‖ Department of Chemistry, Imperial College London, London SW7 2AZ, United Kingdom

**Keywords:** proteomics, cell surface, host-pathogen interaction, infection, neutrophil, metalloprotease, CD55, EHEC, EPHA2, StcE

## Abstract

Enterohemorrhagic *Escherichia coli* (EHEC) is one of several *E. coli* pathotypes that infect the intestinal tract and cause disease. Formation of the characteristic attaching and effacing lesion on the surface of infected cells causes significant remodeling of the host cell surface; however, limited information is available about changes at the protein level. Here we employed plasma membrane profiling, a quantitative cell-surface proteomics technique, to identify host proteins whose cell-surface levels are altered during infection. Using this method, we quantified more than 1100 proteins, 280 of which showed altered cell-surface levels after exposure to EHEC. 22 host proteins were significantly reduced on the surface of infected epithelial cells. These included both known and unknown targets of EHEC infection. The complement decay–accelerating factor cluster of differentiation 55 (CD55) exhibited the greatest reduction in cell-surface levels during infection. We showed by flow cytometry and Western blot analysis that CD55 is cleaved from the cell surface by the EHEC-specific protease StcE and found that StcE-mediated CD55 cleavage results in increased neutrophil adhesion to the apical surface of intestinal epithelial cells. This suggests that StcE alters host epithelial surfaces to depress neutrophil transepithelial migration during infection. This work is the first report of the global manipulation of the epithelial cell surface by a bacterial pathogen and illustrates the power of quantitative cell-surface proteomics in uncovering critical aspects of bacterial infection biology.

## Introduction

Cell-surface proteins are key to the interaction of a cell with its surrounding environment. Among their myriad roles, cell-surface proteins form cell-to-cell contacts (1), control the transport of small molecules (2, 3), and facilitate the sensing of signals and stimuli from other cells (*e.g.* cytokines, hormones) (4). In vertebrates, cell-surface proteins also play a crucial role in the function of the immune system, underpinning the ability to discriminate self from nonself (5), regulating the complement system (6), mediating cell migration (7), and allowing pro- and anti-inflammatory signaling.

Quantitative proteomic analysis has revealed that viral pathogens, such as human immunodeficiency virus (HIV) and human cytomegalovirus, cause significant remodeling of the host cell-surface proteome during infection (8, 9). However, little is known about the manipulation of the host cell surface by bacterial pathogens beyond what has been described for select individual protein targets (10–13).

Here we used plasma membrane profiling (PMP),^5^ a quantitative cell-surface proteomics technique (14), to investigate changes to the host cell surface during enterohemorrhagic *Escherichia coli* (EHEC) infection. This approach identified more than 1100 proteins, 280 of which displayed altered cell-surface levels during infection. Of these proteins, 22 were detected at reduced levels on the surface of infected host cells, suggesting that they are affected by bacterial virulence factors. These proteins include both known and novel targets of bacterial infection.

To validate our analysis, we further examined our top hit. CD55, a key regulator of complement and neutrophil migration, exhibited the greatest reduction at the cell surface during EHEC infection. We show that CD55 is specifically cleaved from intestinal epithelial cells (IECs) by the metalloprotease StcE and demonstrate that CD55 cleavage from the apical surface of IECs results in increased neutrophil attachment to the epithelium.

## Results

### EHEC infection remodels the host cell–surface proteome

**Figure 1.**
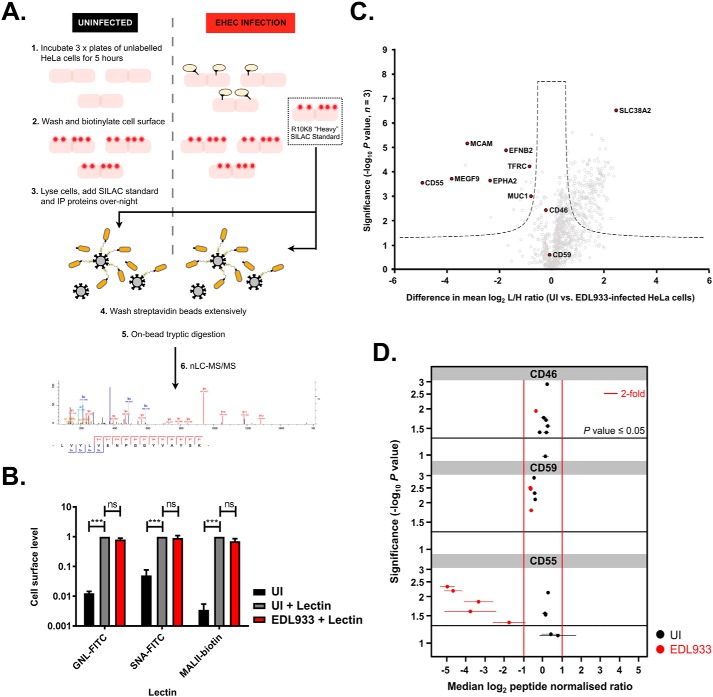
**EHEC infection alters the host cell–surface proteome.** *A*, schematic representation of the experimental setup used for the PMP experiment. Three separate plates of HeLa cells were processed in parallel per test condition (*pink boxes*) (step 1). In addition, uninfected R10K8 “heavy” SILAC HeLa cells were processed to provide an analytical standard (*dotted line box*). After cell-surface biotinylation (step 2), all cells were lysed, protein concentration was determined, and “heavy” SILAC lysates were combined with the test samples (step 3) before immunoprecipitation (step 4), tryptic digestion (step 5), and analysis by nLC-MS/MS (step 6). *B*, cell-surface levels of mannose, α2,6 sialic acid, and α2,3 sialic acid were assessed by flow cytometry using the lectins GNL-FITC, SNA-FITC, and MALII-biotin, respectively. No significant alteration in cell-surface levels of mannose or sialic acids is observed between uninfected (*UI*) or EHEC EDL933–infected cells. Data represent mean ± S.D. of four independent experiments. ***, *p* < 0.001; ns, nonsignificant. *C*, quantitative proteomic analysis of cell-surface proteins after biotinylation and enrichment during EHEC EDL933 infection (5 h). The full proteomics dataset is provided as File S1. A two-sample *t* test was performed (permutation-based FDR = 250, FDR = 0.02, S_0_ = 0.4). *Dashed lines* illustrate the significance cut-off (−log_10_ *p* ≥ 1.3, difference ± 0.7); complement regulatory proteins (CD46, CD55, and CD59) MEGF9, MCAM, EPHA2, EFNB2, MUC1, TFRC and the amino acid transporter SLC38A2 are highlighted in *red. D*, peptide-level *p* value plots for cell-surface complement regulatory proteins. Full peptide-level information is provided as File S2. The probability of differential expression between the uninfected (*UI*) and EHEC EDL933–infected conditions was calculated using Student's *t* test and *p* values were corrected for multiple testing using the Benjamini-Hochberg method. Peptides were deemed significantly affected if the log_2_ ratio was ≥ 1 (2-fold) with a −log_10_ *p* ≥ 1.3. *Error bars* represent 95% confidence intervals. Five unique peptides from CD55 are significantly reduced in EHEC-infected cells compared with uninfected cells, whereas there are no significant differences in abundances of peptides from CD46 and CD59.

Previous work has shown that both EHEC and the closely related pathogen enteropathogenic *E. coli* (EPEC) remove specific proteins from the host cell surface during infection (11–13). To further investigate manipulation of host cell–surface proteins during EHEC infection we performed PMP (14) in conjunction with spike-in SILAC (15), allowing comparison of protein abundances on the surface of uninfected HeLa cells and cells infected with EHEC (Fig. 1*A*). PMP specifically enriches sialylated cell-surface glycoproteins (16). Therefore, we first examined if changes to the glycosylation status of the host cell surface occur during EHEC infection (Fig. 1*B*). No significant changes were observed, suggesting that PMP is a suitable technique for quantitative assessment of cell-surface proteins during EHEC infection.

Comparison of uninfected and EHEC-infected HeLa cells using PMP allowed identification of more than 1100 proteins by at least two unique peptides. Of these proteins, 280 displayed altered cell-surface levels during EHEC infection (−log_10_ *p* ≥ 1.3, difference ± 0.7), 258 being more abundant and 22 less abundant on the surface of infected cells (File S1). The plasma membrane amino acid transporter SLC38A2 (also known as SNAT2) exhibited the greatest increase in cell-surface levels (difference = 2.48) (Fig. 1*C*), likely as a consequence of pathogen-mediated changes to central carbon metabolism (17).

In contrast to SLC38A2, 22 proteins were identified at reduced levels on the host cell surface during infection. This group of proteins included TFRC (difference = −0.83), a receptor that we have previously identified as having reduced cell-surface levels during EHEC infection (13, 18), providing initial validation of our dataset. We thus hypothesized that the remaining 21 proteins may also be involved in processes that are targeted by EHEC virulence factors (Fig. 1*C* and File S1). These proteins include MEGF9 (difference = −3.79), MCAM (also known as MUC18) (difference = −3.21), EPHA2 (difference = −2.34), EFNB2 (difference = −1.73) and MUC1 (difference = −0.76). To test this hypothesis, and further validate our proteomics data, we focused on the protein exhibiting the greatest reduction from the host cell surface during infection, CD55.

**Figure 2.**
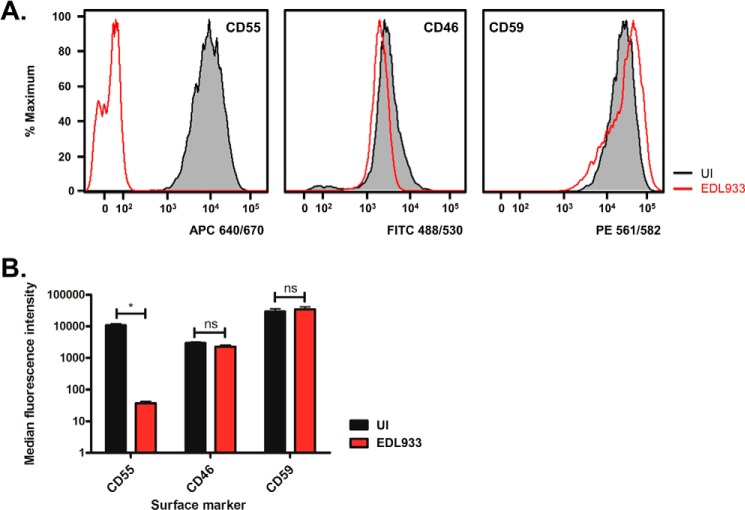
**EHEC infection removes CD55 from the host cell surface.** Flow cytometry was used to quantify the cell-surface levels of CD55, CD46, and CD59 on uninfected (*UI*) and EHEC EDL933–infected HeLa cells. *A* and *B*, representative histograms for each are shown in *A* and quantification from three biological repeats in *B*. Data represent mean ± S.D. *, *p* < 0.05; ns, not significant; two-way analysis of variance (ANOVA) with Sidak multiple comparisons test.

CD55 (difference = −4.93) (Fig. 1*C*) is a cell-surface glycoprotein best characterized as a negative regulator of complement activation. In addition to CD55, two other complement regulatory proteins were identified in our dataset, CD46 and CD59 (5). However, neither of these proteins was significantly affected by EHEC infection at either the whole-protein (Fig. 1*C*, CD46, difference = −0.20; CD59, difference = −0.05) or peptide (Fig. 1*D*) level. Full peptide level information is presented in File S2. We used flow cytometry analysis to quantify the cell-surface levels of CD55, CD46, and CD59 during infection (Fig. 2). Compared with uninfected cells, cells infected with EHEC have a reduced amount of CD55 on their surface, CD46 and CD59 levels are not significantly altered. This confirms the results from the PMP screen and indicates that EHEC specifically removes CD55 from the host cell surface, rather than causing a generalized depression in host protein levels.

### Removal of CD55 from the host cell surface is EHEC-specific and independent of the T3SS

**Figure 3.**
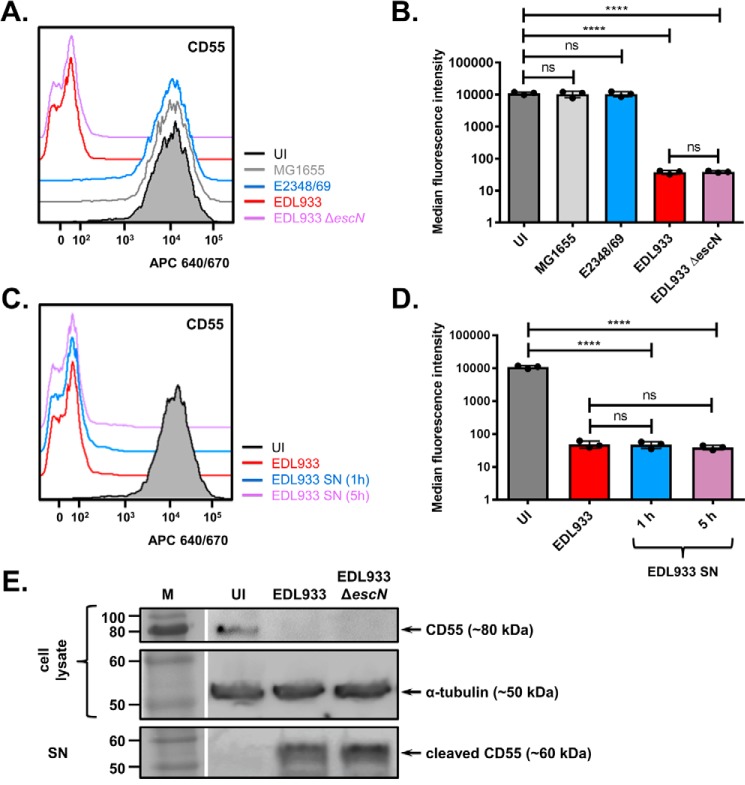
**An EHEC-specific secreted protein cleaves CD55 from the host cell surface.** *A* and *B*, surface levels of CD55 were measured by flow cytometry in uninfected (*UI*) cells and cells infected for 5 h with nonpathogenic *E. coli* (MG1655), EPEC E2348/69, EHEC EDL933, and a T3SS-mutant EHEC strain (EDL933 Δ*escN*). *C* and *D*, surface levels of CD55 were measured by flow cytometry following infection with EHEC EDL933 or treatment with bacteria-free supernatant (*SN*) from EHEC EDL933 cultures for 1 or 5 h. Representative flow cytometry histograms (*A* and *C*) and mean ± S.D. of three biological repeats (*B* and *D*) are shown. ****, *p* < 0.0001; ns, not significant; one-way ANOVA with Tukey's multiple comparisons test. *E*, Western blot analysis of CD55 in the cell lysate containing uncleaved CD55, or in the cell supernatant (*SN*) containing CD55 cleaved from the cell surface. Cells were uninfected (*UI*) or infected with EHEC EDL933 or EHEC EDL933 Δ*escN* for 5 h. Detection of α-tubulin in the cell lysate was used as a loading control. *Gaps* indicate where a lane was removed.

To investigate whether the observed reduction in cell-surface levels of CD55 was because of a specific virulence factor or occurred because of exposure to high numbers of bacteria, we compared the surface levels of CD55 on HeLa cells that were exposed to several *E. coli* strains. These experiments revealed that an EHEC-specific virulence factor was responsible, as neither nonpathogenic *E. coli* nor EPEC was able to reduce surface levels of CD55 (Fig. 3, *A* and *B*).

Given that many EHEC virulence factors use a type three secretion system (T3SS) for delivery, we also tested whether a T3SS mutant (EDL933 Δ*escN*) was able to reduce cell-surface levels of CD55 (Fig. 3, *A* and *B*). This mutant behaved like WT EHEC, indicating that the EHEC-specific virulence factor responsible was not a T3SS effector. Nonetheless, EHEC culture supernatant alone was sufficient to reduce cell-surface levels of CD55, indicating the involvement of a secreted virulence factor (Fig. 3, *C* and *D*). Western blot analysis of HeLa cell culture supernatants and whole cell lysates clearly showed a CD55 cleavage product released from EHEC-infected cells, with concomitant loss of CD55 from cell lysates (Fig. 3*E*). Cumulatively these results suggest that a secreted bacterial protease, specific to EHEC, is likely responsible for cleavage of CD55 from the cell surface during infection. As EHEC produces the factor responsible for the reduction in CD55 levels during growth in DMEM, but not during growth in LB, it is possible that the secreted agent is Ler-regulated (Fig. S1).

### CD55 is removed from epithelial surfaces by the secreted metalloprotease StcE

**Figure 4.**
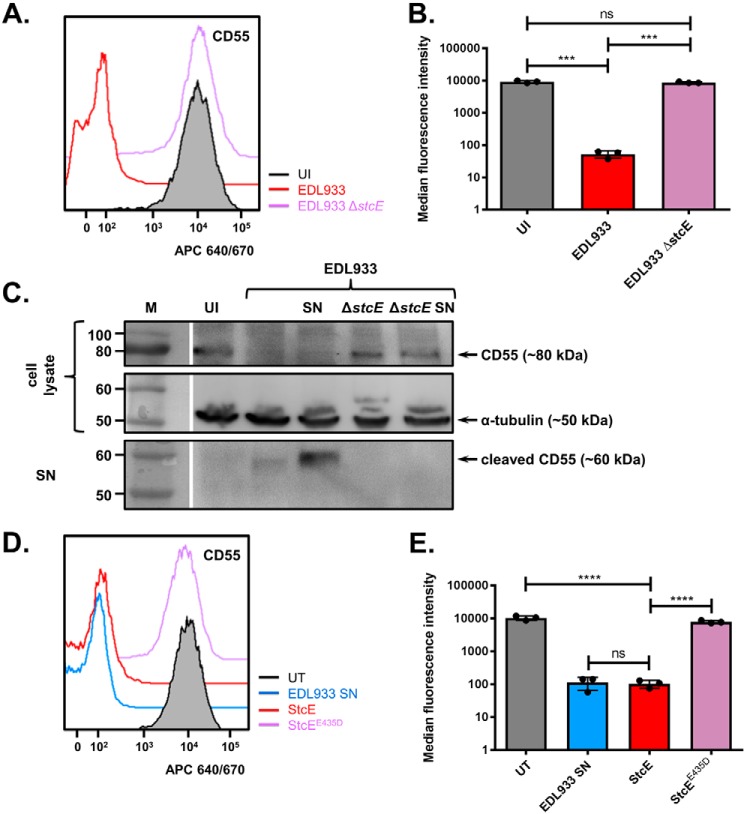
**The EHEC O157:H7 metalloprotease StcE cleaves CD55 from the host cell surface.** *A* and *B*, surface levels of CD55 were measured by flow cytometry on uninfected (*UI*) cells or cells infected with EHEC EDL933 or EHEC EDL933 Δ*stcE. C*, Western blot analysis of CD55 in the cell lysate containing uncleaved CD55 or the cell supernatant (*SN*) containing CD55 cleaved from the cell surface. Cells were uninfected (*UI*), infected with EHEC EDL933 or EHEC EDL933 Δ*stcE* for 5 h, or treated with EHEC EDL933 or EHEC EDL933 Δ*stcE* bacteria-free culture supernatants for 1 h. Detection of α-tubulin in the cell lysate was used as a loading control. *Gaps* indicate where a lane was removed. *D* and *E*, surface levels of CD55 were measured by flow cytometry for untreated (*UT*) cells, cells treated with purified StcE, or cells treated with the catalytically inactive mutant StcE^E435D^. Representative flow cytometry histograms (*A* and *D*) and mean ± S.D. of three biological repeats (*B* and *E*) are shown. ***, *p* < 0.001; ****, *p* < 0.0001; ns, not significant; one-way ANOVA with Tukey's multiple comparisons test.

There are very few EHEC-secreted proteases that are not also encoded by EPEC. We identified two potential candidates from the literature, the autotransporter EspP (19) and the type II–secreted metalloprotease StcE (20); we tested mutants in both proteases. Although EHEC EDL933 Δ*espP* was able to remove CD55 from the host cell surface (Fig. S2), we found that EHEC EDL933 Δ*stcE* was unable to reduce cell-surface levels of this protein (Fig. 4, *A* and *B*). Western blot analysis confirmed that neither infection with EHEC EDL933 Δ*stcE* nor exposure to EHEC EDL933 Δ*stcE* culture supernatants caused CD55 cleavage, as no CD55 was detected in HeLa cell culture supernatants (Fig. 4*C* and Fig. S2).

StcE is a zinc metalloprotease with an active-site glutamic acid at position 435 (20). To confirm that CD55 is cleaved by StcE, we expressed and purified StcE and its inactive catalytic mutant StcE^E435D^ and treated eukaryotic cells with these proteins. WT StcE, but not its catalytic mutant, was able to reduce surface levels of CD55, confirming that the protease activity of StcE is responsible for cleaving CD55 from the host cell surface during infection (Fig. 4, *D* and *E*).

### StcE cleaves CD55 from IECs and reduces neutrophil adhesion

**Figure 5.**
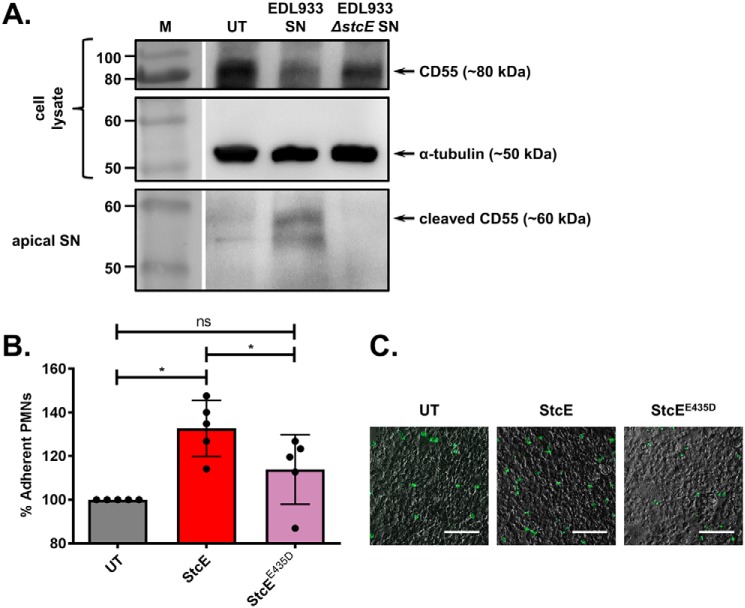
**StcE cleaves CD55 and increases neutrophil retention at the apical surface of intestinal epithelial cells.** *A*, Western blot analysis of CD55 in the cell lysate containing uncleaved CD55 or the apical cell supernatant (*apical SN*) containing CD55 cleaved from the cell surface. Polarized Caco-2 cells were untreated (*UT*) or treated with EHEC EDL933 or EHEC EDL933 Δ*stcE* culture supernatants, which were added to the apical surface of the polarized cells. Detection of α-tubulin in the cell lysate was used as a loading control. *Gaps* indicate where a lane was removed. *B*, neutrophil (PMN) adhesion to Caco-2 monolayers was measured for cells treated with StcE or the catalytically inactive mutant StcE^E435D^ and is expressed as a percentage of the adhesion of PMNs to untreated (*UT*) cells. Data are presented as mean ± S.D. of five independent experiments. *, *p* < 0.05; ns, not significant; one-way ANOVA with Tukey's multiple comparisons test. *C*, untreated, StcE- or StcE^E435D^-treated Caco-2 monolayers with adherent BCECF-AM–loaded neutrophils (*green fluorescence*) were visualized by microscopy. *Scale bars* represent 100 μm.

All experiments shown thus far were performed in HeLa cells as these were used in the original PMP screen. Although HeLa cells are useful for population-wide screening because of the efficiency of EHEC infection (EHEC infects 90–100% of cells), they are not representative of the primary cell target of this pathogen, the apical surface of IECs. CD55 has previously been shown to be present on the apical surface of IECs and this is also true for the colonic IEC line Caco-2 (21, 22). To see if we could recapitulate our results for CD55 with this cell line, the apical surface of polarized Caco-2 monolayers was exposed to EHEC and EHEC Δ*stcE* culture supernatants. Cleaved CD55 was detected in the apical supernatant from IECs exposed to EHEC culture supernatant, but not the apical supernatant from cells exposed to EHEC Δ*stcE* culture supernatant, indicating that StcE is also able to cleave CD55 present on the apical surface of IECs (Fig. 5*A*).

CD55 is best characterized as a complement regulatory factor that reduces the formation of C3 convertase on the cell surface (23). However, it has an additional role as an anti-adhesive factor for neutrophils (otherwise known as polymorphonuclear leukocytes (PMNs)). In this capacity CD55 is responsible for the final step in neutrophil transepithelial migration, the release of neutrophils from the apical surface of IECs into the gut lumen (24). As neutrophil influx is a major mechanism that counters bacterial infection in the gut (25), we tested whether StcE-mediated CD55 cleavage influenced the adhesion of human neutrophils to the apical surface of Caco-2 cells. We found that cells treated with StcE had increased numbers of neutrophils attached after 10 min of adhesion compared with untreated cells or cells treated with the catalytic mutant StcE^E435D^ (Fig. 5, *B* and *C*), providing a complement-independent, biologically relevant function for CD55 cleavage during EHEC infection.

## Discussion

Pathogenic *E. coli* remains a significant cause of gastrointestinal disease throughout the world (26). In the United States alone, multiple outbreaks of EHEC occur annually, often associated with the consumption of contaminated beef products, salad vegetables, and other fresh produce (27). Infection leads to hemorrhagic colitis and, in a subset of cases, hemolytic uremic syndrome, a potentially life-threatening condition characterized by acute renal failure, thrombocytopenia, and hemolytic anemia (27).

EHEC pathogenesis is driven by a number of virulence factors, including a T3SS and a range of effector proteins (many of which are shared by EPEC) (28), the Shiga toxin (which is responsible for hemolytic uremic syndrome) (29), and, in the predominant EHEC strain EHEC O157:H7 (used in this study), the pO157 virulence plasmid (30, 31). Significant attention has been paid to the cytosolic interacting partners of, and the intracellular signaling pathways targeted by, T3SS effectors during EHEC and EPEC infection (28). However, surprisingly little is known about how infection alters the surface of the infected epithelium beyond the formation of the attaching and effacing lesion (32).

In this study we used quantitative cell-surface proteomics (14) to profile the entire host cell–surface proteome during EHEC infection (Fig. 1). Surprisingly, this is one of the first reports of the application of this MS technique during bacterial infection and, to our knowledge, the first using a pathogenic organism (33). We found that EHEC infection of HeLa cells results in altered levels of 280 different host proteins on the host cell surface.

Among proteins with altered cell-surface levels, SLC38A2 (SNAT2) exhibits the greatest increase during EHEC infection (Fig. 1*C*). SLC38A2 is known to mediate uptake of glutamine and other small neutral α-amino acids (2). It therefore plays a role in the feeding of both the tricarboxylic acid (TCA) cycle, as glutamine is readily metabolized to α-ketoglutarate (34), and aerobic glycolysis through the process of glutaminolysis (35). Inhibition of the TCA cycle, and the subsequent switch to aerobic glycolysis, has recently been described during *Citrobacter rodentium* infection of IECs (17). Thus, the increased abundance of SLC38A2 (and its family member SLC38A5 (SNAT5)) (difference = 0.71) (File S1) on the surface of EHEC-infected cells could be a host response to pathogen-mediated changes to central carbon metabolism.

In addition to proteins with increased cell-surface levels, like SLC38A2, we also identified 22 cell-surface proteins that were found at reduced levels during infection (Fig. 1*C* and File S1). These proteins include TFRC, a cell-surface receptor that we have previously shown to be removed from the host cell surface during EHEC infection because of the action of the T3SS effector EspG (13, 18). We therefore suggest that the remaining 21 cell-surface proteins may also be affected by the action of bacterial virulence factors during infection. These proteins include MEGF9, a novel putative receptor protein that is expressed in the intestine and neuronal tissue (36); EPHA2, a cell-surface receptor tyrosine kinase involved in regulation of oxidative stress and inflammation in response to lipopolysaccharide through control of the NF-κB and Nrf2 pathways (37, 38); EFNB2, a membrane-bound ligand of the EPHB1 receptor tyrosine kinase that has been implicated in organization and homeostasis of the gastrointestinal epithelium (39); and the *O-*glycosylated cell-surface mucin MUC1 (40) (File S1). It is interesting to note that EPHA2 has previously been identified as a target during EPEC infection and is phosphorylated in a T3SS-dependent manner (41), further supporting our hypothesis. Whether phosphorylation of EPHA2 by a T3SS effector drives its removal from the cell surface and what role this plays during EHEC/EPEC infection, remain to be determined.

The protein that showed the greatest reduction in our PMP screen was CD55 (Fig. 1*C*), which was almost entirely removed from the host cell surface during EHEC infection (Figs. 1*C* and 2). CD55 is broadly expressed on the surface of human cells, where it functions as a negative regulator of both the classical and alternative complement pathways (5, 23). Removal of CD55 from the cell surface in our experiments is specific, as the cell-surface levels of two other negative regulators of complement, CD46 and CD59, were not significantly altered during EHEC infection (Figs. 1, *C* and *D* and 2). These results validate our proteomics data while also identifying a previously unrecognized host cell target during EHEC infection.

CD55, CD46, and CD59 work in a nonredundant manner to prevent membrane attack complex formation on the cell surface, thus preventing unwanted autologous lysis of “self” cells (5). As CD46 and CD59 were found to be present on the cell surface during EHEC infection (Fig. 2), cells should be protected from membrane attack complex formation, despite the specific cleavage of CD55 by StcE (Figs. 3 and 4). However, patients with loss-of-function mutations in CD55 (CHAPLE syndrome) experience gastrointestinal symptoms (*e.g.* abdominal pain, diarrhea, chronic malabsorption) because of abnormal complement activation (42), suggesting that loss of CD55 alone is sufficient to alter complement activation in the gut. Interestingly, StcE can also recruit the C1 esterase inhibitor (C1-INH) to the cell surface, potentiating its ability to suppress classical complement activation (43). This suggests that StcE can manipulate multiple aspects of the complement regulatory system in the intestine.

Aside from its role in complement regulation, CD55 has also been described as an anti-adhesive molecule that modulates the rate of neutrophil release from the apical surface of IECs (24). To reach the gut lumen neutrophils must first escape the vasculature (transendothelial migration) and then traverse the epithelial layer (transepithelial migration) (44, 45). The terminal step in transepithelial migration is neutrophil release from the apical epithelial surface. CD55 is involved in this process, with down-regulation of CD55 increasing the retention of neutrophils at the apical surface, thus decreasing the overall rate of neutrophil movement across the epithelium (24). To probe the effect of CD55 cleavage by StcE on neutrophil migration, we confirmed that StcE cleaves CD55 from the apical surface of polarized Caco-2 monolayers, the site of both EHEC attachment and CD55 action as an anti-adhesive factor (Fig. 5*A*). Subsequently, we showed that IEC monolayers exposed to WT StcE, but not its catalytic mutant, had increased neutrophil attachment after 10 min of treatment (Fig. 5, *B* and *C*).

The metalloprotease StcE is encoded on the pO157 virulence plasmid in EHEC (31) and is regulated by the master virulence regulator Ler (20, 46, 47). It has been implicated in the cleavage of a number of host glycoproteins, including C1-INH (20) (discussed above) and several mucins (48, 49). The mucinase activity of StcE has been shown to aid clearance of the intestinal mucin layer during EHEC infection (49), and to play a role in epithelial adherence (48). MUC1 was identified as one of the proteins with reduced cell-surface levels in our PMP screen and therefore could also be a target of StcE's mucinase activity (Fig. 1*C* and File S1). In addition to cleaving mucins, StcE acts directly on neutrophils, cleaving CD43 and CD45, and thus altering the neutrophil's adhesive properties (50). The common feature of the StcE substrates identified to date is a heavily *O*-glycosylated N terminus and an EPTT amino acid repeat (48). CD55 shares similar but not identical molecular characteristics, being heavily *O-*glycosylated at its C terminus, rather than N terminus (51), and possessing a tandem QK*X*TT motif within its glycosylation region (Fig. S3). Combined with our data this suggests that StcE acts directly on IECs (through cleavage of CD55) (Figs. 3 and 4) and neutrophils (through cleavage of CD43 and CD45) to control the kinetics of neutrophil infiltration during EHEC infection.

StcE homologs exist in other enteric bacterial pathogens. TagA from *Vibrio cholerae* cleaves CD43 (52), and thus may also regulate neutrophil release from the epithelium, whereas a homologous TagA, found in a diarrheagenic *Aeromonas hydrophila* clinical isolate, cleaves C1-INH in the same way as StcE (53). Although the substrate profiles of these enzymes are not identical to that of StcE (52), it is tempting to speculate that cleavage of CD55 may be a conserved anti-immune mechanism in a subset of enteric bacterial pathogens.

Given the importance of the cell-surface proteome it is not surprising that bacterial pathogens manipulate its composition during infection. Nonetheless, the host cell surface remains a relatively understudied aspect of host–pathogen interaction. In this work we have demonstrated the power and sensitivity of PMP in studying the interplay between bacterial pathogens and their hosts. We have identified both previously known and novel targets of pathogenic *E. coli* infection, proving that this technique provides a robust method to quantify infection-induced changes to the host cell surface that could be applied to many bacterial infection scenarios. In addition, our experiments to validate one of these protein hits, CD55, have revealed new aspects of EHEC host manipulation. The observed changes in neutrophil adhesion at the site of infection, because of CD55 cleavage by StcE (Fig. 5), suggest that this virulence factor, along with clearing host mucins (49), allows EHEC to regulate early inflammatory events at epithelial surfaces and create a favorable environment for replication and transmission.

## Experimental procedures

### Culture of bacterial strains

Unless otherwise stated, all bacterial strains (Table S1) were routinely grown in lysogeny broth (LB) at 37 °C with shaking at 200 rpm. Antibiotic selection was used when necessary as follows: 100 μg/ml ampicillin, 50 μg/ml kanamycin, 25 μg/ml chloramphenicol.

### Maintenance and use of eukaryotic cell lines

HeLa cells (ATCC) were maintained in DMEM with glucose (1 g/liter) supplemented with 10% v/v heat-inactivated FBS (Gibco) and 2 mm GlutaMAX (Invitrogen) at 37 °C, 5% CO_2_. Caco-2/TC-7 cells were maintained in DMEM with glucose (4.5 g/liter) supplemented with 15% v/v FBS, 2 mm GlutaMAX, and 0.1 mm nonessential amino acids at 37 °C, 10% CO_2_. Cell lines were routinely passaged using 0.1% w/v trypsin/0.02% w/v EDTA (Gibco).

For R10K8 stable isotope labeling with amino acids in cell culture (SILAC), HeLa cells were grown in ready-to-use R10K8 DMEM (1 g/liter glucose) containing ^13^C- and ^15^N-labeled arginine and lysine (Dundee Cell Products) supplemented with dialyzed heat-inactivated FBS (ultrafiltration against 0.15 m NaCl, 10,000-Da cut-off) (Sigma). Cells were grown for seven passages before storage at −80 °C. When thawed, cells were maintained and expanded in the same media before use. The R10 and K8 incorporation rates were determined to be >98% by MS analysis.

Cells were seeded as necessary prior to each experiment. HeLa cells were seeded in 96-well plates at a density of 1 × 10^4^ cells per well, in 6-well plates at 2 × 10^5^ cells per well, and in T90 1-well plates at 1 × 10^6^ cells per plate. Caco-2 cells were seeded in 6.5 mm Transwell^®^-COL permeable supports with collagen-coated 3.0 μm PTFE membranes (Corning) or 96-well plates at a density of 2 × 10^4^ cells per well. The culture medium was changed on a daily basis for 7–14 days after the formation of a confluent monolayer. Confluence was monitored by measurement of transepithelial electrical resistance using an EVOM epithelial voltmeter (World Precision Instruments).

### Infection assays

EHEC EDL933 and *E. coli* K-12 were grown in LB for 8 h before subculture (1:1000) in DMEM (1 g/liter glucose) for 16–18 h, 37 °C, 5% CO_2_. Stationary phase cultures were diluted 1:20 in DMEM (1 g/liter glucose) before addition to cells at a multiplicity of infection (m.o.i.) of ∼100:1. Plates were centrifuged at 500 × *g* for 5 min to synchronize infection. Infected cells were incubated for 2.5 h at 37 °C, 5% CO_2_ before washing three times with PBS and addition of fresh DMEM (1 g/liter glucose + 100 μg/ml gentamicin). Cells were incubated for a further 2.5 h.

EPEC E2348/69 was grown in LB overnight before subculture (1:100) in DMEM (1 g/liter glucose) and incubation for 3 h at 37 °C, 5% CO_2_. Bacteria were subsequently added to cells at a multiplicity of infection of ∼100:1. Infected cells were incubated at 37 °C, 5% CO_2_ for a total of 2 h. After 1 h cells were washed with PBS (three times) and fresh media was added.

Bacterial supernatants were obtained by filtering overnight DMEM cultures of EHEC through a 0.2 μm Acrodisc® syringe filter with Supor® membrane (Pall) before 1:20 dilution and addition to cells. Cells were incubated for 1–5 h with the bacterial supernatant. For assays performed on polarized Caco-2 cells, bacterial supernatants were added to the apical side of cells and incubated for 1 h. Purified protein (StcE or StcE^E435D^) in DMEM was added to cells at 0.1 μg protein per well before cells were incubated for 1 h at 37 °C, 5% CO_2_.

### Cell-surface biotinylation and sample preparation for MS-based quantitative proteomics

HeLa cells were seeded 48 h prior to infection in T90 1-well plates and infected with EHEC when necessary. Monolayers were washed with pre-warmed PBS (twice) and chilled PBS (once). Surface sialic acid residues were oxidized by addition of 1 mm sodium metaperiodate on ice for 30 min. All subsequent steps were performed at 4 °C. Oxidized cells were washed with PBS (once) before addition of 6 ml biotinylation solution (250 μm aminooxy-biotin (Biotium), 10 mm aniline, ice-cold PBS, pH 6.7) and incubated in the dark for 30 min, with gentle agitation. Biotinylation reactions were quenched by washing with 1 mm glycerol in ice-cold PBS, pH 6.7, before further washing with PBS + 5% v/v FBS, pH 6.7, and PBS, pH 6.7. As needed, T90 plates of R10K8 SILAC-labeled HeLa cells were grown in parallel to provide spike-in material. Three cell-surface enrichment experiments were performed in parallel for each condition.

Biotinylated cells were lysed (lysis buffer: 150 mm NaCl, 1% v/v Triton X-100, 50 mm Tris-HCl, pH 7.6, 5 mm iodoacetamide, 0.1 mg/ml phenylmethanesulfonyl fluoride, supplemented with cOmplete mini protease inhibitor tablets (Roche)) and scraped into 1.8 ml microtubes before incubation at 4 °C for 30 min with end-over-end mixing followed by centrifugation at 2800 × *g* for 10 min. Supernatants were retained and centrifuged twice at 16,000 × *g* for 10 min. Protein concentrations were determined using the Pierce BCA protein assay kit (Thermo Fisher Scientific), and 0.3 mg of infected, light-labeled lysate was mixed with R10K8-labeled lysate at a ratio of 2:1.

Biotinylated proteins were immunoprecipitated from lysate mixtures using high capacity Streptavidin Agarose beads (Pierce) overnight at 4 °C with end-over-end mixing. Beads were washed extensively (Lysis Buffer (20 times), PBS + 0.5% w/v SDS (20 times), 6 m urea + 100 mm Tris-HCl pH 8.5 (40 times), 5 m NaCl (20 times), 100 mm sodium carbonate (20 times), PBS (20 times), dH_2_O (20 times), 50 mm ammonium bicarbonate (AMBIC) (5 times)) using a vacuum manifold and snap-cap spin columns (Pierce). Samples were incubated for 5 min with end-over-end rotation between each solute. Samples contained in a 40 μl bed of beads were reduced by addition of 4 μl 100 mm dithiothreitol (DTT) in 50 mm AMBIC at 55 °C for 30 min and allowed to cool to room temperature. Beads were washed with 50 mm AMBIC (twice) before addition of 4 μl 100 mm iodoacetamide in 50 mm AMBIC at room temperature for 30 min (in the dark) before washing with 50 mm AMBIC (twice). 1.6 μg of trypsin (modified sequencing grade, Promega) dissolved in 50 mm AMBIC was added to the beads and samples were digested overnight at 37 °C with shaking at 1000 rpm. Samples were centrifuged and the supernatant transferred to clean LoBind tubes (Eppendorf). Beads were washed with 0.1% v/v formic acid in dH_2_O and washes were combined with the original supernatant fractions. Solutions were purified using StageTips as described previously (54) and peptides eluted from the sorbent (SDB-XC Poly(styrene/divinylbenzene) Copolymer) with 79% v/v acetonitrile in dH_2_O, followed by SpeedVac-assisted solvent removal and storage at −80 °C. Peptides were reconstituted in 0.5% v/v TFA in dH_2_O and transferred to LC-MS sample vials for injection.

### nLC-MS/MS analysis

Analysis of peptide mixtures was carried out using an Acclaim^TM^ PepMap^TM^ RSLC column with an inner diameter of 50 cm × 75 μm (Thermo Fisher Scientific) using a 2-hour acetonitrile gradient in 0.1% v/v formic acid in dH_2_O, flow rate of 250 nl/min and an Easy-nLC 1000 coupled to a Q Exactive Hybrid Quadrupole-Orbitrap^TM^ Mass Spectrometer via an EASY-Spray nano-ESI source (all Thermo Fisher Scientific). The Q Exactive Hybrid Quadrupole-Orbitrap^TM^ Mass Spectrometer was run in data-dependent mode with survey scans acquired at a resolution of 75,000 at *m*/*z* 200. Up to 10 of the highest abundance isotope patterns with a charge of +2 or higher were selected to proceed from the survey scan (isolation window of *m*/*z* 3.0) to fragmentation by higher-energy collision dissociation with normalized collision energies of 25. Maximum ion injection times for the survey scan and the MS/MS scan (resolution of 17,500 at *m*/*z* 200) were 20 and 120 ms, respectively. Ion target value for MS was 10^6^ and for MS/MS was 10^5^. The intensity threshold was 8.3 × 10^2^.

### Mass spectrometry data and processing

The MS proteomics data have been deposited to the ProteomeXchange Consortium via the PRIDE partner repository with the dataset identifier PXD010361.

### MaxQuant analysis

Data were processed using MaxQuant version 1.5.8.5 (55). Peptides were identified from MS/MS spectra searched against the UniProt *Homo sapiens* reference proteome (proteome ID: UP000005640) and the UniProt EHEC O157:H7 EDL933 reference proteome (proteome ID: UP000028484) using the Andromeda search engine (56). Cysteine carbamidomethylation was specified as a fixed modification and methionine oxidation and N-terminal acetylation were selected as variable modifications. *In silico* digests of reference proteomes were performed using the Trypsin/P setting with up to three missed cleavages allowed. The false discovery rate (FDR) was set at 0.01 for peptides, proteins, and sites. Both the “re-quantify” and “match between runs” functions were enabled. The sequence decoy mode used was “revert.” All other parameters were used as pre-set in MaxQuant.

### Perseus analysis

Data were analyzed using Perseus version 1.5.0.9 (57). Proteins present in the “reverse,” “only identified by site,” and “potential contaminant” databases were removed, and proteins identified by two or more unique peptides retained for further analysis. Normalized heavy to light ratios were transformed and values were logarithmized (log_2_). The three replicates for each biological condition were grouped together and at least two valid values in each group were required for further comparison. Volcano plots were generated using log_2_ light/heavy ratios for comparison of each biological condition, an FDR of 0.02, and S_0_ of 0.4. Further analysis of the SILAC data at the peptide level was performed using a custom pipeline development in Pipeline Pilot (Biovia) as described previously (58).

GOCC terms (59) used to identify cell-surface proteins were as follows: anchored to plasma membrane, plasma membrane, integral to plasma membrane, intrinsic to plasma membrane, cell surface, cell projection membrane, extracellular region, apical plasma membrane, basal plasma membrane, and external side of plasma membrane.

### Protein expression, purification, and characterization

*E. coli* SHuffle T7 cells (New England Biolabs) were transformed with plasmids pTB4 and pTB5 (Table S1) expressing StcE and its catalytically inactive variant StcE^E435D^, respectively (43). In both constructs the signal sequence targeting the protein to the periplasm was omitted and proteins were expressed with C-terminal His_6_ tags. Transformants were initially grown on LB agar plates supplemented with 50 μg/ml kanamycin, subsequently used to inoculate small-scale overnight cultures (5 ml LB) in 50 ml polypropylene tubes, supplemented with 25 μg/ml kanamycin, and incubated at 37 °C with shaking at 200 rpm for 15–18 h. 500 ml of LB in 2.5 liter flasks, supplemented with 25 μg/ml kanamycin, were inoculated from the overnight cultures (1:250) and the resulting suspensions were incubated at 37 °C with shaking at 200 rpm until *A*_600_ 0.8. 1 mm isopropyl β-d-1-thiogalactopyranoside (IPTG) was used for protein induction and cultures were further grown at 25 °C with shaking at 200 rpm for 15 h. Disruption of the cells was performed by sonication followed by centrifugation at 16,000 × *g* for 40 min at 4 °C.

After isolation, each cell lysate was applied onto 10 ml of Fast Flow Chelating Sepharose (Amersham Biosciences) charged with Ni^2+^ and equilibrated with 50 mm Tris-HCl, pH 7.5, 300 mm NaCl, 20 mm imidazole. The column was washed with the equilibration buffer, followed by 4 column volumes of 50 mm Tris-HCl, pH 7.5, 300 mm NaCl, 100 mm imidazole. Proteins were eluted with 50 mm Tris-HCl, pH 7.5, 300 mm NaCl, 200 mm imidazole and eluents were exchanged into 50 mm Tris-HCl, pH 7.5, 150 mm NaCl and concentrated (30,000-Da cut-off). Protein concentration was determined using the Pierce BCA reducing agent compatible protein assay kit (Thermo Fisher Scientific) and samples analyzed by SDS-PAGE for purity.

### Flow cytometry

Following infection, treatment with bacterial supernatants or treatment with purified protein, eukaryotic cells were washed with PBS (twice) and harvested using Cell Stripper (Corning). Cells were blocked with 2% v/v FBS in PBS and labeled with fluorophore-conjugated primary antibodies against CD55, CD46, and CD59. For cells labeled with biotinylated *Maackia amurensis* agglutinin II (MALII-biotin), *Galanthus nivalis* agglutinin-FITC (GNL-FITC), or *Sambucus nigra* agglutinin-FITC (SNA-FITC), lectins were diluted in lectin-binding buffer (0.15 m NaCl, 10 mm HEPES, pH 7.5, PBS) before addition to cells. When necessary, Streptavidin-RRX (Life Technologies) was used at a dilution of 1:800 in PBS to detect biotinylated lectins. All incubations were for 45 min on ice. Labeled cells were fixed with 4% v/v paraformaldehyde in PBS for 10 min, washed with PBS, resuspended in 300 μl of PBS, and filtered prior to analysis.

Flow cytometry was performed on a four-laser, 13-color Fortessa cytometer (BD Biosciences). Experiments were performed in technical duplicate with 10,000 events analyzed per sample. Data were analyzed using FlowJo^TM^ 10 (FlowJo LLC).

### Western immunoblotting

Following infection, host cell supernatants were collected, precipitated using 10% v/v TCA, and washed with ice-cold acetone twice before resuspension in 2× Laemmli buffer (from 5× stock, 312.5 mm Tris-HCl, pH 6.8, 10% w/v SDS, 50% v/v glycerol, 0.5 m DTT, 0.05% w/v bromphenol blue). Infected cells were lysed with 1× Laemmli buffer and cell lysates collected on ice. All samples were boiled for 5 min prior to SDS-PAGE analysis and Western blotting. Proteins were transferred to Hybond-P PVDF membranes (GE Healthcare) using a Trans-Blot Turbo semi-dry transfer system (Bio-Rad). Membranes were blocked with 5% w/v skimmed milk in PBS with 0.1% v/v Tween 20 (PBS-T) prior to addition of primary antibodies, rabbit anti-CD55, 1:1000 (Thermo Fisher Scientific) and mouse anti-α-tubulin, 1:2000 (Sigma). HRP-conjugated secondary antibodies (Jackson ImmunoResearch Laboratories) were used when necessary (goat anti-rabbit-HRP, 1:5000 and goat anti-mouse-HRP, 1:2000). Membranes were washed three times 5 min with PBS-T prior to development with EZ-ECL chemiluminescence reagent (Geneflow). Blots were visualized using a LAS-3000 Imager (Fujifilm). For re-blotting, blots were stripped using stripping buffer (15 mg/ml glycine, 0.5% w/v SDS, 1.0% v/v Tween 20, pH 2.2).

### Neutrophil adhesion assay

Neutrophils (PMNs) were isolated from whole blood obtained from healthy human volunteers using PolymorphPrep according to manufacturer's instructions. Ethical approval for drawing and using human blood was provided by the Regional Ethics committee and the Imperial NHS trust tissue bank (REC Wales approval: 12/WA/0196, ICHTB HTA license: 12275) and abides by the Declaration of Helsinki principles. Isolated PMNs were resuspended in Hanks' Balanced Salt Solution (HBSS) with 10 mm HEPES (pH 7.4) and without Ca^2+^ or Mg^2+^ (HBSS−) and labeled for 30 min at 37 °C with 5 μm 2′,7′-bis(2-carboxyethyl)-5-(and -6)-carboxyfluorescein acetoxymethyl ester (BCECF-AM) (Life Technologies). Then, they were washed in HBSS− and resuspended in HBSS with 10 mm HEPES (pH 7.4) and Ca^2+^ or Mg^2+^ (HBSS+) and used within 2 h of isolation.

Caco-2 monolayers were treated with recombinant StcE or StcE^E435D^ at 0.1 μg per 10^5^ cells for 1 h at 37 °C, 5% CO_2_. Cells were washed and 100 nm fMLF (Sigma) in HBSS+ was added for 10 min at 37 °C, 5% CO_2_ before addition of 1 × 10^5^ labeled PMNs. Plates were centrifuged at 100 × *g* for 5 min to settle the PMNs and adhesion was allowed to occur for 10 min at 37 °C, 5% CO_2_. Monolayers were then washed gently with HBSS+ and fluorescence intensity (excitation at 485 nm; emission at 530 nm) was measured using an Infinite M200 Pro microplate reader (Tecan). Adherent PMNs were quantified by generating standard curves of BCECF-AM–labeled PMNs.

### Immunofluorescence microscopy

Cells were fixed with 4% v/v paraformaldehyde in PBS for 10 min before addition of 50 mm NH_4_Cl. Monolayers were visualized using an Axio Observer Z1 widefield epifluorescence microscope (Zeiss).

## Author contributions

R. C. D. F., L. F. D., and A. C. formal analysis; R. C. D. F. validation; R. C. D. F., W. W. L., D. A. I. M., L. F. D., and A. C. investigation; R. C. D. F., W. W. L., L. F. D., and A. C. visualization; R. C. D. F., D. A. I. M., L. F. D., A. I. W., and A. C. methodology; R. C. D. F. and A. C. writing-original draft; R. C. D. F., D. A. I. M., and A. C. writing-review and editing; A. I. W. and E. T. resources; A. I. W. and E. T. software; A. I. W. and A. C. supervision; E. T. and A. C. project administration; A. C. conceptualization; A. C. data curation.

## Supplementary Material

Supporting Information

## Footnotes

5 The abbreviations used are:

PMP: plasma membrane profiling
EHEC: enterohemorrhagic *Escherichia coli*
CD55: cluster of differentiation 55
IEC: intestinal epithelial cell
EPEC: enteropathogenic *E. coli*
SILAC: stable isotope labeling with amino acids in cell culture
T3SS: type three secretion system
Caco-2 cells: human epithelial colorectal adenocarcinoma cells
PMN: polymorphonuclear leukocyte (neutrophil)
TCA: tricarboxylic acid
AMBIC: ammonium bicarbonate
FDR: false discovery rate
PBS-T: PBS with 0.1% v/v Tween 20
HBSS: Hanks' Balanced Salt Solution
BCECF-AM: 2′,7′-bis(2-carboxyethyl)-5-(and -6)-carboxyfluorescein acetoxymethyl ester
MALII-biotin: biotinylated *Maackia amurensis* agglutinin II
GNL-FITC: *Galanthus nivalis* agglutinin-FITC
SNA-FITC: *Sambucus nigra* agglutinin-FITC
ANOVA: analysis of variance.
