# Supplementary material for: Plasma membrane profiling during enterohemorrhagic E. coli infection reveals that the metalloprotease StcE cleaves CD55 from host epithelial surfaces
Source: J Biol Chem. 2018 Sep 6;293(44):17188–99. doi: 10.1074/jbc.RA118.005114 (PMC6222108; doi:10.1074/jbc.RA118.005114)
Supplement: Supporting Information [file supp_293_44_17188__index.html]

Plasma membrane profiling during enterohemorrhagic E. coli infection reveals that the metalloprotease StcE cleaves CD55 from host epithelial surfaces — EHEC removes CD55 from epithelial surfaces — Plasma membrane profiling during enterohemorrhagic E. coli infection reveals that the metalloprotease StcE cleaves CD55 from host epithelial surfaces — EHEC removes CD55 from epithelial surfaces — Supporting Information 

# Plasma membrane profiling during enterohemorrhagic *E. coli* infection reveals that the metalloprotease StcE cleaves CD55 from host epithelial surfaces

## Supporting Information

- Supporting Information (to be published online) - Supporting Information text
- Supporting Information (to be published online) - File S1-Proteomics data
- Supporting Information (to be published online) - File S2-Peptides
