# Supplementary material for: Plasma membrane profiling during enterohemorrhagic E. coli infection reveals that the metalloprotease StcE cleaves CD55 from host epithelial surfaces
Source: J Biol Chem. 2018 Sep 6;293(44):17188–99. doi: 10.1074/jbc.RA118.005114 (PMC6222108; doi:10.1074/jbc.RA118.005114)
Supplement: Supporting Information [file supp_RA118.005114_139760_1_supp_197936_pdmwzt.pdf]

SUPPORTING INFORMATION

Plasma membrane profiling during enterohemorrhagic *E. coli* infection reveals that the metalloprotease StcE cleaves CD55 from host epithelial surfaces

R. Christopher D. Furniss[1,†], Wen Wen Low[1,†], Despoina A.I. Mavridou1, Laura F. Dagley[2,3], Andrew I. Webb[2,3], Edward W. Tate[4], Abigail Clements1[,*]

From the [1]MRC Centre for Molecular Bacteriology and Infection, Department of Life Sciences, Imperial College London, London, United Kingdom; [2]Walter and Eliza Hall Institute of Medical Research, Melbourne, Australia; [3]Department of Medical Biology, University of Melbourne, Melbourne, Australia; [4]Department of Chemistry, Imperial College London, London, United Kingdom

[†]These authors contributed equally to this work

[*]**To whom correspondence should to be addressed:** A. Clements: MRC Centre for Molecular Bacteriology and Infection, Department of Life Sciences, Imperial College London, London SW7 2AZ; a.clements@imperial.ac.uk

This PDF file includes:

Figure S1	Ler-inducing conditions are required for cleavage of CD55
Figure S2	EspP does not cleave CD55
Figure S3	Schematic representation of CD55
Table S1	Bacterial strains used in this work
File S1 legend	Comprehensive cell-surface proteome dataset for EHEC EDL933 infection
File S2 legend	Comprehensive peptides.txt output from MaxQuant

SUPPORTING FIGURES

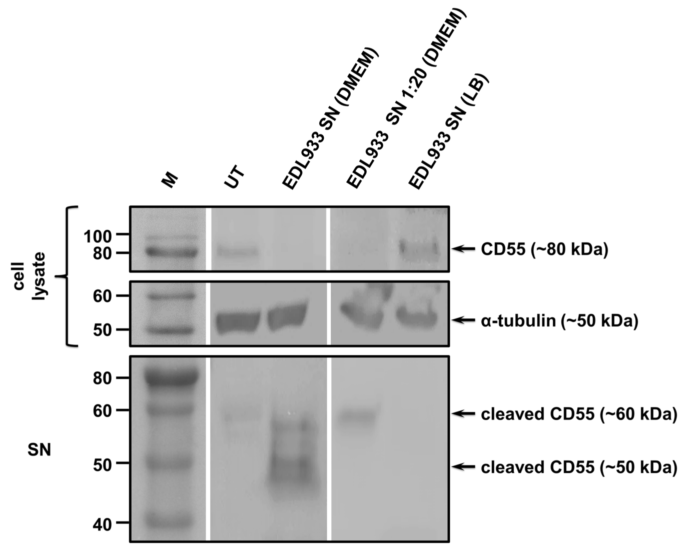

Figure S1. Ler-inducing conditions are required for cleavage of CD55. Western blot analysis of CD55 in the cell lysate containing uncleaved CD55 or the cell supernatant (SN) containing CD55 cleaved from the cell surface. Cells were untreated (UT) or treated with bacteria-free supernatant from EHEC EDL933 cultures grown in Ler-inducing conditions (DMEM) or non-inducing conditions (LB). Supernatants were added to cells neat or at a 1:20 dilution (equivalent to MOI 100:1, used for infection) as indicated. Detection of α-tubulin in the cell lysate was used as a loading control. Gaps indicate where a lane was removed.

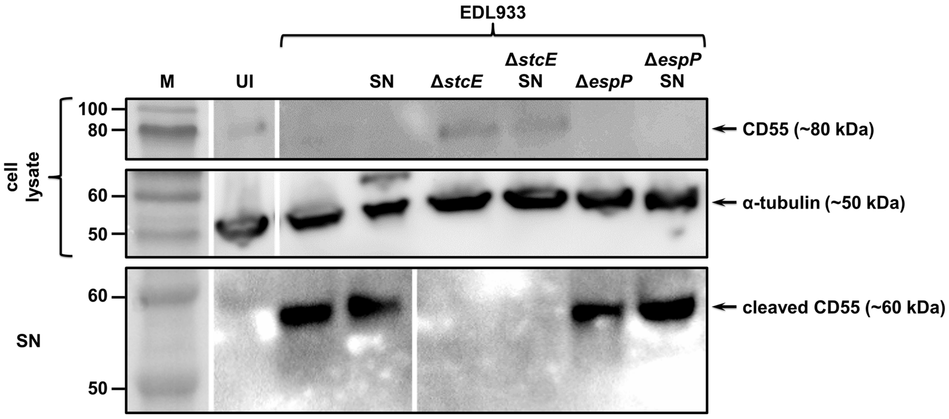

Figure S2. EspP does not cleave CD55. Western blot analysis of CD55 in the cell lysate containing uncleaved CD55 or the cell supernatant (SN) containing CD55 cleaved from the cell surface. Cells were uninfected (UI), infected with EHEC EDL933, EHEC EDL933 Δ*stcE* or EHEC EDL933 Δ*espP* for 5 hours, or treated with EHEC EDL933, EHEC EDL933 Δ*stcE* or EHEC EDL933 Δ*espP* bacteria-free culture supernatants for 1 hour. Detection of α -tubulin in the cell lysate was used as a loading control. Gaps indicate where a lane was removed.

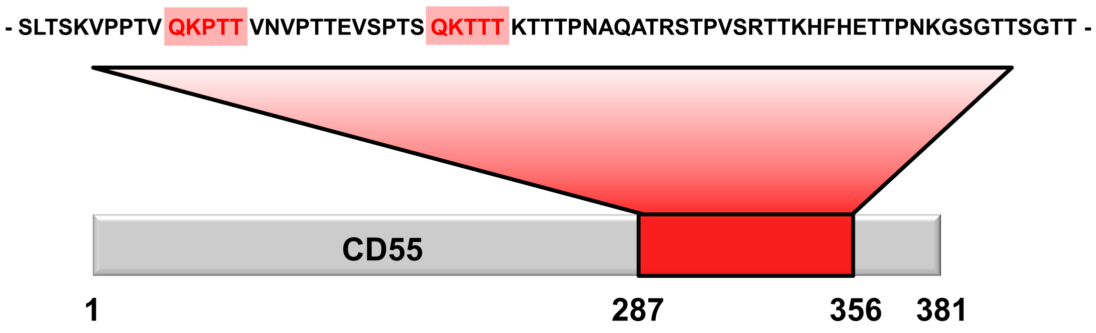

Figure S3. Schematic representation of CD55. The red-highlighted section represents the C-terminal serine/threonine-rich region (aa 287-356) that is heavily O-glycosylated. The position of the Q-K-X-T-T repeat motifs (aa 297-301 and aa 314-318) within this region are indicated in red.

SUPPORTING TABLES

Table S1. Bacterial strains used in this work

Strain	Description	Source
Enterohaemorrhagic *E. coli* (EHEC) O157:H7 EDL933	wild type (stx1-/stx2-)	(1)
EHEC EDL933 Δ*escN*	Δ*escN::Kn*, T3SS mutant	(2)
EHEC EDL933 Δ*stcE*	Δ*stcE::Cm,* deletion mutant	(3)
EHEC EDL933 Δ*espP*	Δ*espP*::*Kn,* deletion mutant	(4)
E. coli MG1655	K-12, non-pathogenic	ATCC 700926
Enteropathogenic *E. coli* (EPEC) O127:H6 E2348/69	wild type	(5)
E. coli SHuffle® T7	expression strain	New England BioLabs
E. coli SHuffle® pTB4	$StcE^{24\text{-}886}$ in pET24d(+), C-terminal His_6-tag	(6)
E. coli SHuffle® pTB5	$StcE^{24\text{-}886}$ in pET24d(+), E435D mutation, C-terminal His_6-tag	(6)

LEGENDS FOR SUPPORTING FILES

File S1. Results of the plasma membrane profiling experiment for EHEC EDL933 infection. The file contains the comprehensive quantitative proteomics dataset, detailing proteins with altered abundance on the cell surface (CS). On the “Full CS Dataset” tab, significantly changing proteins are indicated with a “+” in the “Significant change” column. In the “Reduced on CS” tab, significantly changing proteins are indicated with a “+” in the “Significant change” column and highlighted in green. In the “Increased on CS” tab, significantly changing proteins are indicated with a “+” in the “Significant change” column and highlighted in green. In all tabs, proteins marked in orange are proteins originating from the infecting EHEC EDL933 bacteria.

File S2. Peptides.txt MaxQuant output. Comprehensive peptides.txt output from MaxQuant detailing peptide-level information used to generate Fig. 1D.

REFERENCES

1. Riley, L. W., Remis, R. S., Helgerson, S. D., McGee, H. B., Wells, J. G., Davis, B. R., Hebert, R. J., Olcott, E. S., Johnson, L. M., Hargrett, N. T., Blake, P. A., and Cohen, M. L. (1983) Hemorrhagic colitis associated with a rare Escherichia coli serotype. *N. Engl. J. Med.* **308**, 681-685
2. Garmendia, J., Phillips, A. D., Carlier, M. F., Chong, Y., Schuller, S., Marches, O., Dahan, S., Oswald, E., Shaw, R. K., Knutton, S., and Frankel, G. (2004) TccP is an enterohaemorrhagic Escherichia coli O157:H7 type III effector protein that couples Tir to the actin-cytoskeleton. *Cell. Microbiol.* **6**, 1167-1183
3. Lathem, W. W., Grys, T. E., Witowski, S. E., Torres, A. G., Kaper, J. B., Tarr, P. I., and Welch, R. A. (2002) StcE, a metalloprotease secreted by Escherichia coli O157:H7, specifically cleaves C1 esterase inhibitor. *Mol. Microbiol.* **45**, 277-288
4. Xicohtencatl-Cortes, J., Saldana, Z., Deng, W., Castaneda, E., Freer, E., Tarr, P. I., Finlay, B. B., Puente, J. L., and Giron, J. A. (2010) Bacterial macroscopic rope-like fibers with cytopathic and adhesive properties. *J. Biol. Chem.* **285**, 32336-32342
5. Levine, M. M., Bergquist, E. J., Nalin, D. R., Waterman, D. H., Hornick, R. B., Young, C. R., and Sotman, S. (1978) Escherichia coli strains that cause diarrhoea but do not produce heat-labile or heat-stable enterotoxins and are non-invasive. *Lancet* **1**, 1119-1122
6. Lathem, W. W., Bergsbaken, T., and Welch, R. A. (2004) Potentiation of C1 esterase inhibitor by StcE, a metalloprotease secreted by Escherichia coli O157:H7. *J. Exp. Med.* **199**, 1077-1087
